# Effectiveness of hemostatic agents in thyroid surgery for the prevention of postoperative bleeding

**DOI:** 10.1038/s41598-020-58666-4

**Published:** 2020-02-04

**Authors:** Martino Scaroni, Urs von Holzen, Christian A. Nebiker

**Affiliations:** 1 Department of Anesthesiology, Cantonal Hospital of Grisons, Löestrasse 170, 7000 Chur, Switzerland; 2 Indiana University School of Medicine South Bend, Goshen Center for Cancer Care, 200 High Park Ave., Goshen, IN 46526 USA; 3 Department of General Surgery, Cantonal Hospital of Aarau, Tellstrasse, Haus 3, 5000 Aarau, Switzerland

**Keywords:** Thyroid gland, Thyroid diseases

## Abstract

Postoperative bleeding remains one of the most frequent, but rarely life-threatening complications in thyroid surgery. Although arterial bleeding is the main cause of postoperative hemorrhage, most often no actively bleeding vessel can be found during revision. Therefore, the coagulation technique for larger vessels may play a minor role, and hemostatic agents could be of higher importance. In this descriptive, retrospective study, data of 279 patients with thyroid surgery (total of 414 thyroid lobectomies) were collected. We reviewed the electronic medical record by analyzing the histological, operative, laboratory and discharge reports in regards to postoperative bleeding. Of the 414 operated thyroid lobes, 2.4% (n = 10) bled. 1.4% (n = 6) needed reoperation while the other 1.0% (n = 4) could be treated conservatively. Hemostatic patches were applied 286 (69.1%) times. Of the 128 (30.9%) patch-free operated sides, 4.7% (n = 6) suffered postoperative bleeding. Tachosil® alone was used 211 (51.0%) times and bleeding occurred in 1.4% (n = 3). Without statistical significance (p = 0.08) the use of Tachosil® seems to help preventing postoperative bleeding. The combination with other patches doesn’t appear to be more efficient.

## Introduction

Due to its endocrine function, the thyroid gland is a well perfused organ. For this reason, a good hemostasis during surgical procedures is of utmost importance. Postoperative bleeding remains one of the most frequent complications, and can cause airway compression and respiratory distress in severe cases. Moreover, intraoperative bleeding can obscure important structures like the parathyroid glands and the recurrent laryngeal nerve, complicating surgical dissection and increasing morbidity Aleksandar, *et al*.^1–3^. The frequency of postoperative cervical hematoma (PCH) in thyroid surgery requiring reoperation ranges from 0 to 9.1%^4^, and it is the most common reason for return to the operating room^5^.

Many factors influence perioperative hemostasis. In the literature, several preoperative patient’s risk factors are described, such as Grave’s disease^6–9^, benign pathology^6,8,9^, the use of anticoagulation or antiplatelet medication while doing surgery^6,10^, an increased size of the pathology specimen^6^, an emergent admission^8^, presence of 2 or more comorbidities^8^, previous thyroid surgery^9^, age 65 years and older^7,11^, African-American race^7^ and history of alcohol abuse^7^. A BMI greater than 30 kg/m^2^ ^11,12^ and male sex^7,9,11^ are also independent risk factors associated with PCH. Furthermore, postoperative hypertension increases the risk of postoperative bleeding^9,13^.

In the past century, the only instruments at a surgeon’s disposal to obliterate vessels were needle and thread in order to create a ligation. Nowadays, the industry provides a variety of devices to seal and cut vessels, such as metal clips, bipolar coagulation forceps (e.g. BiClamp®) and ultrasound scissors. The type of sealing technique doesn’t seem to affect the risk of postoperative bleeding. Alesina *et al*. and Brzezinski *et al*. found no difference in complication rate by using BiClamp® or ligations in their studies. Other vessel sealing systems, like LigaSure® and Harmonic Scalpel® have proven to be efficient in safely sealing the vessels with minimal thermal effect on the surrounding tissue, but they are expensive and only meant for a single use^14,15^. On the other hand, many studies such as the one from McNally *et al*. showed no difference in comparing ultrasonic dissection devices (Harmonic Focus®) with electronic vessel sealing systems (LigaSure®) in several criteria except from operating time, which was shorter for ultrasonic devices^16^.

Although arterial bleeding is the main cause of postoperative hemorrhage, as it starts earlier than venous oozing. Most often no actively bleeding vessel can be found during reoperation^12^. Therefore, the coagulation technique for the larger vessel may play a minor role. As certain conditions, like for example the need of anticoagulant agents cannot be changed for different reasons, hemostatic agents may play an important role. Therefore, we focused on sealant patches.

Basically, there are two main categories of hemostatic patches available: biologically active and physical agents. The first group consists of sealant matrixes containing human coagulation factors as a coating on the surface. In contact with body fluids, such as blood, the components of the coating diffuse into the wound activating the fibrinogen-thrombin reaction, thus initiating the last phase of the human coagulation cascade. This process leads to the formation of a fibrin clot, which holds the collagen matrix to the wound surface, creating a mechanically stable network that provides sealing. Among the commercially available products are Surgiflo®, Floseal® (gelatin-thrombin matrix sealants), Evarrest® and Tachosil® (fibrinogen and thrombin coated matrix), the latter being most frequently used at the University Hospital of Basel (USB). The second group consists of physical agents that enhance hemostasis using a passive substrate. An oxidized cellulose patch, when saturated with blood, provides a surface for platelet adhesion and aggregation, thus initiating the formation of a durable clot that doesn’t wash away or bleed again when irrigated. Tabotamp Snow®, Spongostan™ and Nu-Knit® are examples that belong to this type of physical agents.

The use of hemostatic agents in thyroid surgery has been widely reported in the literature, but their effectiveness in preventing postoperative bleeding remains controversial^17^. Considering biologically active agents only, some studies demonstrated an advantage over standard treatment in terms of mean operation time, reduction of 24-h drain output, time to drain removal, incidence of post-operative seroma and length of hospital stay^1,3,4,18–22^. Others found a lack of advantages on reducing perioperative bleeding, time to drain removal and length of hospital stay^23,24^, causing only additional costs^24^. Furthermore, Testini *et al*. and Scerrino *et al*. found that both biologically active and physical hemostatic agents reduced the mean operation time and the 24-h drain output, with the first ones being more effective^3,21^. However, physical agents didn’t show advantages over conventional surgical techniques on time to drain removal, length of hospital stay and seroma formation^3,4,21^. We found no studies proving physical agents being more efficient than biologically active ones^1,3,4,21^. Only Amit *et al*. found that the use of an oxidized cellulose hemostatic agent resulted in longer time to drain removal, and consequently in length of hospital stay, if compared with conventional procedures, suggesting that postoperative hemorrhage can best be prevented by proper intraoperative hemostasis^25^.

Although delayed absorption (normally it takes 6 weeks) of oxidized cellulose may mimic an abscess or a tumor, which can lead to unnecessary aspiration attempts, the use of the hemostatic agents appears to be safe^26^. According to Ujam *et al*. there is no association between the use of Floseal®, and the occurrence of adverse effects such as allergic reaction, wound breakdown, wound infection and thrombosis^27^. Furthermore, the use of an oxidized cellulose patch was not associated with a higher incidence of wound infection, hypocalcemia or recurrent laryngeal nerve palsy^25,28^.

The aim of our study was to investigate the correlation between the use of hemostatic patches and the incidence of postoperative bleeding.

## Methods

### Study design and patient population

In this retrospective, descriptive study, we analyzed 279 consecutive patients who underwent thyroid surgery at the University Hospital of Basel (USB) in Switzerland between 2007 and 2012.

Patients above the age of 18 years requiring thyroid surgery were included. We excluded three patients who had surgery because of parathyroid gland disease, one patient who underwent surgery due to a cyst in the neck and one patient who had neck exploration with lymphadenectomy of the central cervical compartment.

The whole study was conducted in accordance with the Declaration of Helsinki, and the protocol was approved by the local ethic committee.

### Data collection

We reviewed each patient’s electronic medical records by analyzing the histological, operative, laboratory and discharge reports. Parameters included in our database were: operation date, sex, hormonal status (hypothyroidism, euthyroidism, hyperthyroidism), substitution with L-Thyroxin, thyreostatic therapy, presence of Graves’ disease, ultrasound finding, diameter of the biggest thyroid nodule, availability of FNA, cytology finding in FNA (Bethesda criteria if available), definitive histology, thyroid scintigraphy (if performed), presence of thyroiditis, number of operated sides, surgical procedure, intention of surgical procedure, operation time, combined parathyroidectomy, lymphadenectomy, devices used for dissecting the upper pole vessels, intraoperative neuromonitoring before and after resection, the use of hemostatic agents, presence of anticoagulation, placing of a drain, postoperative hypocalcaemia, treating of hypocalcaemia, recurrent laryngeal nerve palsy, course of laryngeal nerve palsy, postoperative bleeding and impairment of wound healing.

### Compliance with ethical standards

The study was performed in accordance with the ethical standards of the institutional and National Research Committee (“Ethikkommission beider Basel EKBB, Nr. 2013/132) and with the 1964 Helsinki declaration and its later amendments or comparable ethical standards.

For this retrospective analysis no written informed consent was requested by the National Research Committee.

### Study end points

The end point of our study was postoperative bleeding. Although we collected data to audit additional topics, such as hypocalcaemia and recurrent laryngeal nerve palsy, we concentrated on postoperative bleeding. We clinically evaluated the occurrence of a neck hematoma. We provided information on the necessity to treat the hemorrhage with a second operation.

### Statistical analysis

The data were entered into an Excel spreadsheet (Microsoft Office®, Version 2010). Statistical analysis was conducted using R version 3.1.2 (R Foundation for Statistical Computing, Vienna, Austria).

## Results

### Baseline characteristics and clinical presentation

The baseline characteristics of the 279 investigated patients, the clinical presentation and the use of anticoagulant and/or antiplatelet medication are summarized in Tables 1 and 2.

**Table 1:** Baseline characteristics and clinical presentation.

Patients, n	279		
Mean age, years	52 ± 15		
Sex	Female, n (%)	Male, n (%)	
	193 (69.2)	86 (30.8)	
Mean operation time, min	137		
Operated sides	One side, n	Two sides, n	Isolated isthmectomy, n
(n = 414)	140	137	2
Thyroid function	Euthyroidism, n (%)	Hypothyroidism, n (%)	Hyperthyroidism, n (%)
	222 (79.6)	22 (7.9)	35 (12.5)
Medication	L-Thyroxin, n (%)	Thyrostatic agent, n (%)	
	25 (9)	32 (11.5)

**Table 2:** Anticoagulant and /or antiplatelet medication.

	Patients	Operated lobes
Aspirin®, n (%)	22 (59.5)	34 (58.6)
Plavix®, n (%)	2 (5.4)	3 (5.3)
Marcoumar®, n (%)	7 (18.9)	9 (15.5)
Low-molecular-weight-heparin (LMWH), n (%)	3 (8.1)	6 (10.4)
Aspirin® + Plavix®, n (%)	1 (2.7)	2 (3.4)
Plavix® + LMWH, n (%)	1 (2.7)	2 (3.4)
Marcoumar® + LMWH, n (%)	1 (2.7)	2 (3.4)
Total, n (%)	37 (3.3)	58 (14)

### Postoperative bleeding

Of the 414 operated thyroid lobes, 2.4% (n = 10) experienced postoperative bleeding. 1.4% (n = 6) needed a second surgical procedure, while the other 1.0% (n = 4) could be treated conservatively. Data regarding the cases with postoperative bleeding, the device used to obliterate the upper pole vessels, and the application of hemostatic patches are summarized in Tables 3 and 4.

**Table 3:** Postoperative bleeding with regard to used device to close the upper pole vessels and application of hemostatic patches.

	Conservative treated postoperative bleeding	Reoperated postoperative bleeding
Number, n (%)	4 (1.0)	6 (1.4)
Device/Technique		
Ligation, n	3	3
BiClamp® and metal clips, n	1	2
Unknown, n		1
Hemostatic patch		
Tachosil®		3
Tachosil® and Tabotamp Snow®	1	
No hemostatic patch	3	3

**Table 4:** Hemostatic patches and postoperative bleeding.

	Operated lobes	Postoperative bleeding
No hemostatic patches, n (%)	128 (30.9)	6 (4.7)
Tachosil® alone, n (%)	211 (51.0)	3 (1.4)
Other hemostatic patch, n (%)	75 (18.1)	1 (0.24)

For the 414 thyroid lobectomies, 4 different instruments were used alone, or sometimes in combination to obliterate the upper pole vessels. Ligation was used in 55.8% (n = 231) of the cases, metal clips in only 2.4% (n = 10), BiClamp® in 28.5% (n = 118), ultrasound scissors in 0.5% (n = 2) and in 12.8% (n = 53) unfortunately the sealing device was not documented.

For the 6 patients that underwent reoperation due to postoperative bleeding, 3 times ligation, and twice BiClamp® combined with metal clips were used. For one patient it is unknown how the upper pole vessels were ligated. Regarding the 4 conservatively treated hemorrhages, in 3 cases ligation was used, and in the remaining patient (25%, n = 1) BiClamp® was used in combination with metal clips.

Hemostatic patches were applied on 286 (69.1%) of the 414 thyroid lobectomies performed. Tachosil® was used 211 (73.8%) times, Tabotamp Snow® 47 times (16.4%), Spongostan™ 6 times (2.1%), Tachosil® and Spongostan™ together 7 times (2.4%), Tachosil® and Tabotamp Snow® together 10 times (3.5%), and Nu-Knit® 5 times (1.8%).

Of the 6 patients who underwent reoperation for postoperative bleeding, Tachosil® was applied 3 times. In the other 50% of the cases, there were no hemostatic patches. For the 4 cases with conservative management of bleeding, once the combination of Tachosil® and Tabotamp Snow® was applied, and 3 times no hemostatic patches were used.

Of the 128 (30.9%) operated sides without hemostyptic patches applied, 4.7% (n = 6) suffered postoperative bleeding. Tachosil® alone was used 211 (51.0%) times and bleeding occurred in 1.4% (n = 3) of the cases. Although the difference in the rate of postoperative bleeding was not significant (Fisher’s exact test: p = 0.08), there was a trend to less bleedings when Tachosil® was used. Other hemostatic patches (even in combination with Tachosil®) were used in 18.1% (n = 75) and postoperative hemorrhage occurred once (0.24%).

Among the 37 patients under anticoagulation and/or antiplatelet medication, postoperative bleeding occurred in 5.4% (n = 2) of the cases, or in 6.9% (n = 4) if related to the 58 thyroid lobectomies performed. One patient was under Aspirin®, the other one was taking Plavix®. Both patients needed to be reoperated. None of them received a hemostatic patch at the time of the initial operation.

70.3% (n = 26) of the anticoagulated patients received a hemostyptic patch. The most used was Tachosil® with 65.4% (n = 17), followed by Tabotamp Snow® (19.3%, n = 5), Nu-Knit® (7.7%, n = 2), Tachosil® + Tabotamp Snow® (3.8%, n = 1) and Tachosil® + Spongostan® (3.8%, n = 1). In regard to operated sides, hemostyptic agents were used in 74.1% of the cases (n = 43), divided as follow: Tachosil® 67.3% (n = 29), Tabotamp Snow® (18.6%, n = 8), Nu-Knit® (4.7%, n = 2), Tachosil® + Tabotamp Snow® (4.7%, n = 2) and Tachosil® + Spongostan® (4.7%, n = 2).

## Discussion

Complications of thyroid surgery decreased dramatically over the last few decades. However, our study showed that even the newest techniques and devices didn’t eliminate them completely.

The rate of postoperative bleeding in our patient cohort (2.4%) is consistent with the described rates in the literature (0 to 9.1%)^4^.

Our study further suggests that hemostatic patches are helping in the prevention of postoperative bleeding. When no hemostatic patch was applied, we could observe a hemorrhage frequency of 4.7%. The use of Tachosil® (not combined with another hemostyptic agent) reduced the rate to 1.4%, even though this difference did not reach statistical significance in the Fisher’s exact test (p = 0.087). The combination of Tachosil® with other patches did not appear to be more efficient, as in this case bleeding occurred in 1.8% of the cases.

Erdas *et al*. focused on the effectiveness of Tachosil® in patients taking Aspirin® or vitamin K antagonists (VKA). In this patient’s subgroup, 7.1% developed a PCH, a relative high percentage. When only patients under VKA were considered, the rate reached 14.8%. However, in their study, the use of Tachosil® has not proven to be more effective than the standard hemostasis techniques in terms of postoperative bleeding, time to drain removal and length of hospital stay. Indeed, of the 5 patient who bled after surgery, 3 (60%) received Tachosil®^23^. Thirty-seven (13.3%) patients of our cohort were taking antiplatelet and/or anticoagulation drugs. Similar to Erdas *et al*., we observed an increased risk of PCH in this patient subgroup. Among them, 2 (5.4%) developed a postoperative bleeding requiring a second operation. If we correlate these data to the number of operated sides, we see a bleeding rate increase to 6.9% (4 of 58 operated sides). Both of these patients were taking an antiplatelet drug (Aspirin®, respectively Plavix®), and none of them was treated with Tachosil® or got another hemostyptic patch. Although none of the patients of our cohort under antiplatelet and/or anticoagulation drugs treated with Tachosil® or other hemostyptic patches had postoperative bleeding, this patient’s subgroup maybe profits more than others from the application of sealant patches. Further studies with larger cohorts are needed.

At the beginning of the study period, ligations were used primarily to seal the upper pole vessels. Later on, BiClamp® became our standard sealing instrument, and to provide even higher safety, were almost always combined with metal clips. This shift towards the bipolar clamp was also supported by Alesina *et al*. and Brzezinski *et al*.. In their study, they found no difference in complication rates by using BiClamp® or ligations. Other vessel sealing systems, like LigaSure® and Harmonic Scalpel®, have proven to be efficient in safely sealing the vessels with minimal thermal effect on the surrounding tissue, but they are expensive and only meant for a single use^14,15^. In our cohort, postoperative bleeding occurred approximately with the same frequency in the ligation and the BiClamp® group.

A number of limitations of this study should be addressed. The investigated cohort is relatively small. A bigger sample size would probably have achieved statistical significance. The retrospective nature didn’t allow us to do a stratification of our study population in a case-matches way and that led to the presence of several confounding factors and bias. The studied patient population results to be heterogeneous for pre-operative risk factors (such as the regular intake of anti-platelet and/or anticoagulation drugs), pathologies and procedures performed. Furthermore, some of the investigated instruments were introduced at the USB only after the collection of data started, and they were therefore not available for the whole study population. The anticoagulation medication, the type of vessel sealing system, and the hemostyptic patch may all have an influence on the postoperative bleeding rate, but due to the small sample size this became not obvious.

## Conclusion

Our study demonstrated that the application of hemostatic patches, and the use of modern devices such as the BiClamp® are safe and may be helpful in preventing postoperative bleeding. Our cohort showed a trend to less postoperative bleeding by using hemostyptic patches, although missing significance. A larger randomized trial would be necessary to definitively answer the question whether hemostatic agents should be used in thyroid lobectomy to prevent postoperative bleeding.
